# Effect of ZnO:Cs_2_CO_3_ on the performance of organic photovoltaics

**DOI:** 10.1186/1556-276X-9-323

**Published:** 2014-06-27

**Authors:** Hyeong Pil Kim, Abd Rashid bin Mohd Yusoff, Hee Jae Lee, Seung Joo Lee, Hyo Min Kim, Gi Jun Seo, Jun Ho Youn, Jin Jang

**Affiliations:** 1 Department of Information Display and Advanced Display Research Center, Kyung Hee University, Dongdaemun-ku, Seoul 130-171, Republic of Korea

**Keywords:** Polymer solar cells, Inverted structure, ZnO, Cs_2_CO_3_, PEDOT, PSS

## Abstract

We demonstrate a new solution-processed electron transport layer (ETL), zinc oxide doped with cesium carbonate (ZnO:Cs_2_CO_3_), for achieving organic photovoltaics (OPVs) with good operational stability at ambient air. An OPV employing the ZnO:Cs_2_CO_3_ ETL exhibits a fill factor of 62%, an open circuit voltage of 0.90 V, and a short circuit current density of −6.14 mA/cm^2^ along with 3.43% power conversion efficiency. The device demonstrated air stability for a period over 4 weeks. In addition, we also studied the device structure dependence on the performance of organic photovoltaics. Thus, we conclude that ZnO:Cs_2_CO_3_ ETL could be employed in a suitable architecture to achieve high-performance OPV.

## Background

The performance of organic solar cells significantly improved during the last few years. Both industrial and academic sectors have focused on the enhancement of their performance, developed new materials, and also improved the stability of the devices. Organic solar cells have attracted a huge interest, given that they are easy to make on flexible substrates, using roll-to-roll technology [1-4], which significantly reduces the manufacturing costs [5,6].

Although we have seen a significant improvement in the performance of organic solar cells, the efficiency of organic solar cells is still far behind their counterparts, inorganic solar cells. Organic solar cells are basically fabricated by sandwiching a photoactive layer between two electrodes. Normally, in the conventional device architecture, a poly (3,4-ethylenedioxythiophene):poly (styrenesulfonate) (PEDOT:PSS) layer is employed as an anode buffer layer [7-9]. However, one major drawback of using PEDOT:PSS is its poor stability.

Therefore, another alternative to avoid the use of PEDOT:PSS is to make use of an inverted structure [10-22], where the anode and cathode positions are reversed, and *n*-type metal-oxide-semiconductors, namely, ZnO, TiO_*x*_, AZO, and NiO_*x*_, are used [2-5], instead of the PEDOT:PSS. Despite device architecture, there is another factor which one can consider in order to enhance the performance of optoelectronic devices, which is the energy barrier between layers. One may find that by decreasing this energy barrier, charge carrier injection at the interface can be significantly improved and therefore, device performance can be improved [23-26]. To date, various methods have been introduced to tune the work functions between semiconductors and metals such as plasma treatment, absorption of atoms, and also the introduction of additional thin-films [27-31].

Zinc oxide (ZnO) has attracted considerable interest for its optical, electrical, and mechanical properties. Experimental and theoretical studies on ZnO crystals have revealed the presence of a permanent dipole moment, which yields a significant piezoelectric effect for a variety of mircomechanical devices. ZnO has been shown to be a good electron selective and hole blocking contact in inverted solar cells. The conduction band (CB) and valence band (VB) of ZnO have been reported to be −4.4 and −7.8 eV, respectively [15]. This allows ZnO to function as a good interfacial layer between ITO and the bulk-heterojunction blend for inverted solar cell devices. ZnO also has large exciton binding energy of about 60 meV, which has been shown to be valuable for optoelectronic devices such as light-emitting diodes and lasers. Nevertheless, ZnO has one major drawback, which is the lack of stable and reproducible *p*-type ZnO with low resistivity, high carrier concentration, and high carrier mobility.

Doping with the first group elements like Li, Na, K, and Cs in ZnO would substitute Zn^2+^ by the monovalent cations, thus making it possible to realize *n*-type conduction. The realization of *n*-type conduction is very important for ZnO applications in optoelectronic devices, and there are reports on the electrical property of the first group element-doped ZnO thin-films [32-36]. Various techniques such as pulsed laser deposition [37,38], magnetron sputtering [39,40], and molecular beam epitaxy [41] have been used to deposit thin-films of ZnO. The sol-gel method [42] has been receiving increased attention because of its many advantages such as low cost, simple deposition procedure, easier composition control, low processing temperature, and easier fabrication of large area films. Therefore, here, we demonstrate the improved performance of P3HT:PCBM and P3HT:ICBA-based inverted bulk-heterojunction solar cells through the appropriate interface modification by Cs_2_CO_3_-doped ZnO on the electron collecting ITO interface. Recently, Yang *et al*. has reported that a solution-processed Cs_2_CO_3_ is able to make interface dipoles layer on ITO. One may say that these two entities (ZnO and Cs_2_CO_3_) are completely different but the most important thing is that these entities do improve the performance of the device. Moreover, we have seen a number of works on tuning the work function of ITO by adding an electron transport layer such as ZnO [43], TiO_2_[44-46], Cs_2_CO_3_[44-46], and poly(ethylene oxide) (PEO) [47]. The created dipole moment helps to reduce the work function of ITO, allowing ITO to serve as the cathode. The improved device performance is due to the reduction of series resistance, improved shunt performance, and enhanced open-circuit voltage of the cell which can be attributed to the improvement of the following aspects: (1) reduction of the contact resistance between the ZnO:Cs_2_CO_3_ and active organic layer, (2) enhancement of the electronic coupling between inorganic ZnO:Cs_2_CO_3_ and active organic layer to mediate better forward charge transfer and reduce back charge recombination at the interface, and (3) affect the upper organic layer growth mode and morphology.

## Methods

### ZnO solution preparation

ZnO solution was prepared using similar procedures to the one reported by Jang *et al*. [27]. Cs_2_CO_3_ solution was prepared by dissolving in ethanol in the ratio of 1.25 wt%.

### Organic solar cell fabrication

**Figure 1:**
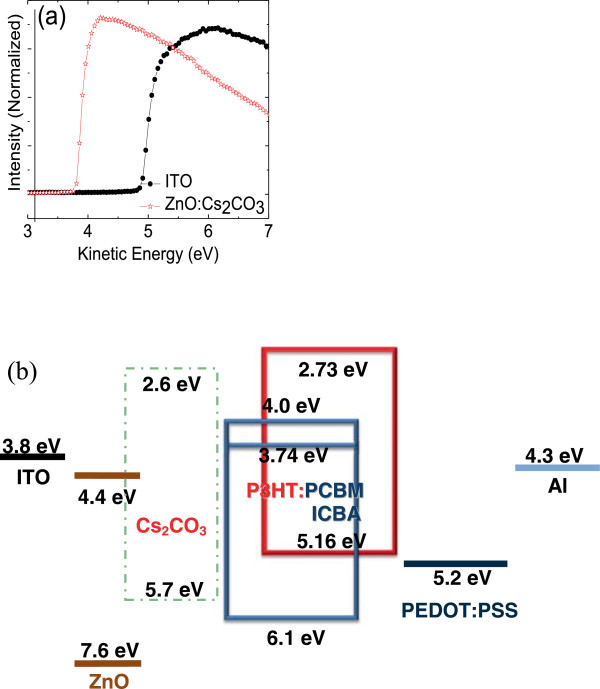
**Detailed values extracted from the UPS spectra and schematic diagram of organic solar cells. (a)** Evolution of secondary electron edge of ITO and ITO/ZnOCs_2_CO_3_ and **(b)** energy level alignment of all materials used in this study.

Schematic diagram of organic solar cells is shown in Figure 1b, where the device is fabricated using pre-patterned ITO-coated glass substrate. Prior to the use, the substrate was cleaned in ultrasonic using acetone, methanol, and isopropanol, rinsed with deionized water, and later dried with N_2_ compressor. All cleaned substrates were treated with UV Ozone treatment for 15 min.

The solution for electron selective layer was prepared by mixing ZnO and Cs_2_CO_3_ with different blend ratios, namely, 1:1, 1:2, 1:3, 2:1, and 3:1. The solution-processed ZnO or ZnO:Cs_2_CO_3_ was spin-coated at 1,000 rpm for 25 s onto the cleaned substrates and later annealed at 300°C for 10 min. The photoactive layer either P3HT:PCBM or P3HT:ICBA dissolved in 1,2-dichlorobenzene was spin-coated at 700 rpm for 25 s and subsequently annealed at 130°C for 30 min or 150°C for 10 min, respectively. Later, PEDOT:PSS was spin-coated at 4,000 rpm for 25 s onto the photoactive layer and annealed at 120°C for 20 min. To complete the device, 100-nm thick of Al was thermally evaporated at rates 4 A/s through a shadow mask at a base pressure of 10^−7^ Torr. The active area of the complete devices is 0.04 cm^2^. To ensure the reproducibility of our results, we have fabricated 83 devices throughout this work.

The following are the fabricated devices based on different photoactive materials.

*P3HT:PCBM-based devices*.

Device A-ITO/ZnO/P3HT:PCBM/PEDOT:PSS/Al

Device B-ITO/ZnO:Cs_2_CO_3_/P3HT:PCBM/PEDOT:2PSS/Al

*P3HT:ICBA-based devices*.

Device C-ITO/ZnO/P3HT:ICBA/PEDOT:PSS/Al

Device D-ITO/ZnO:Cs_2_CO_3_/P3HT:ICBA/PEDOT:PSS/Al

### Thin film and device characterizations

The *J-V* characteristics of the conventional solar cells were measured using the Keithley 2400 source meter under a solar simulator (AM1.5) with an irradiation intensity of 100 mW/cm^2^.

The EQE measurements were performed using an EQE system (Model 74000) obtained from Newport Oriel Instruments, Irvine, CA, USA, and the HAMAMATSU calibrated silicon cell photodiode (HAMAMATSU, Shizuoka, Japan) was used as the reference diode. The wavelength was controlled with a monochromator to range from 200 to 1,600 nm.

AFM imaging was achieved in air using a Digital Instrument Multimode that is equipped with a nanoscope IIIa controller.

XPS measurements were performed in a PHI 5000 VersaProbe (Ulvac-PHI, Chigasaki, Kanagawa, Japan) with background pressure of 6.7 × 10^−8^ Pa, using a monochromatized Al Kα (*hv* = 1,486.6 eV) anode (25 W, 15 kV).

Ultraviolet photoemission spectroscopy (UPS) measurements were carried out using the He 1 photon line (*hv* = 21.22 eV) of a He discharge lamp under UHV conditions (4 × 10^−10^ mbar).

The transmittances of ZnO, and ZnO:Cs_2_CO_3_ coated on ITO-glass substrates were recorded at room temperature with a SCINCO S4100 (SCINCO, Seoul, South Korea) spectrophotometer.

XRD measurements were carried out using X'PERT PRO of PANalytical Diffractometer (PANalytical, Seongnam City, South Korea) with a Cu Kα source (wavelength of 1.5405 Å) at 40 kV and 100 mA and at a speed of 1°/min.

Raman scattering experiments were performed at room temperature using a Ramanor T-64000 microscopy system (Jobin Yvon, Longjumean, France).

Photoluminescence (PL) spectra were recorded using a lock-in technique with JASCO FP-6500 (JASCO, Easton, MD, USA)composed of two monochromators for excitation and emission, a 150-Watt Xe lamp with shielded lamp house and a photomultiplier as light detector.

## Results and discussion

### i-XPS

**Figure 2:**
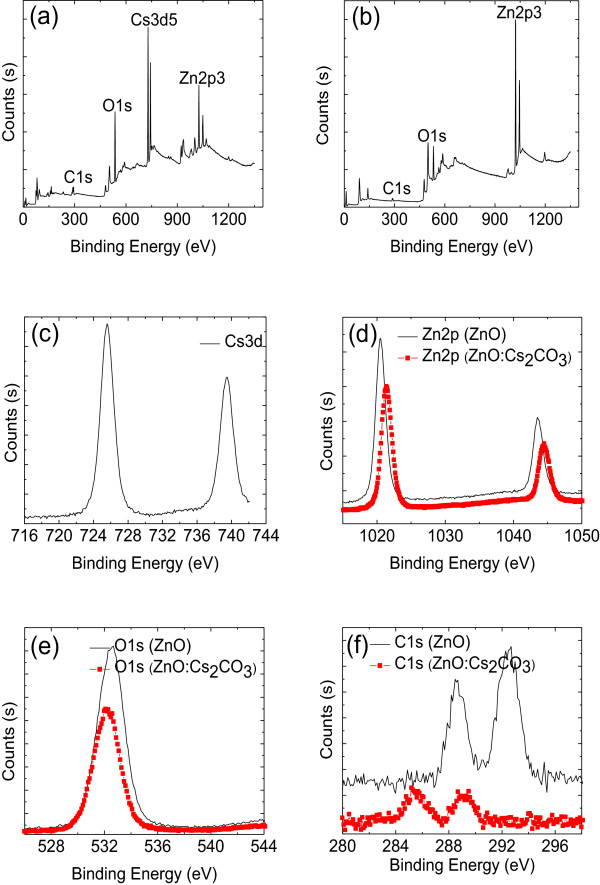
**The XPS spectra of ITO/ZnO and ITO/ZnO:Cs**_**2**_**CO**_**3 **_**films.** XPS survey spectra of **(a)** ZnO:Cs_2_CO_3,_**(b)** ZnO, high-resolution XPS spectra of **(c)** Cs, **(d)** Zn, **(e)** O, and **(f)** C of Cs_2_CO_3_-doped ZnO thin film coated on Si wafer.

The XPS spectra of ITO/ZnO and ITO/ZnO:Cs_2_CO_3_ films are shown in Figure 2. It can be seen that the O 1 s and C 1 s binding energies shift to lower level after the deposition of 20 nm ZnO:Cs_2_CO_3_ film on ITO compared to that of bare ITO/ZnO. Meanwhile, the Zn 2p peak of the 20-nm-thick ZnO:Cs_2_CO_3_ film keeps higher binding energy compared to that of the 20-nm-thick ITO/ZnO film. Furthermore, the reaction between ITO and Cs_2_CO_3_ may also originated from the Sn or In-O-Cs complex [48], which further lowers the work function of ITO. As for the XPS spectra, the realization of the ZnO:Cs_2_CO_3_ interfacial layer remarkably reduces the electron injection barrier from ITO. It is generally known that interface modification by doping results in the enhancement of electron injection due to the reduction of the electron injection barrier [48-51]. One possible reason is that during evaporation, Cs_2_CO_3_ tends to decompose into two different compounds, CsO_2_ and CO_2_, to form a X-O-Cs complex, consequently increasing the electron injection [48]. In addition, the metallic compound Cs is diffused into the ZnO surface to form an efficient electron injection contact during the thermal evaporation of Cs_2_CO_3_[50]. Moreover, the improvement of free-electron density can also be considered to be one of the main factors in the increment of electron injection [51].

### ii-UPS and contact angle

In order to clarify the advantage of the ZnO:Cs_2_CO_3_ as the interfacial layer, the effect of ZnO:Cs_2_CO_3_ on interfacial layer properties is investigated by UPS. As shown in UPS spectra (Figure 1a), the work function of ITO is determined to be 4.7 eV, and upon the interface modification, the work function of ITO decreased to 3.8 eV. We interpret this decrease in work function as arising from the interfacial dipoles from the modified ZnO:Cs_2_CO_3_ layer, which reduces the vacuum level, resulting in a lower electron injection barrier, thus facilitating electron injection [48]. Therefore, the establishment of the interfacial dipole or interface modification induces lower work function of ITO, which may reduce the electron-injection barrier height compared to the case without interface modification. The detailed values extracted from the UPS spectra are shown in Figure 1a. As depicted from the energy diagram shown in Figure 1b, the electron injection barrier from ITO to ZnO:Cs_2_CO_3_ is reduced from 2.1 to 1.2 eV. Obviously, this cathode interface modification greatly reduces the electron injection barrier, which should be beneficial for the improvement of PCE. The complete structure of our inverted organic solar cells is shown in Figure 1b. The interface modification was also carried out by taking multiple contact angle measurements from few locations on the substrates, with and without interface modification. Contact angle measurements were performed to confirm that interface modification was present on the ITO film. Six separate contact angle determinations were performed on each sample. Without interface modification, the surface of ZnO after oxygen plasma had a low wetting angle to DI water (~26°) - showing a hydrophilic (oleophobic) surface. It is worth noting that such a low contact angle indicates a higher surface energy, which is characteristic for polar surfaces. The creation of the interface modification layer was confirmed from the data, which demonstrates the enhancement in contact angle (hydrophobic/oleophilic surface) after surface modification (~68°).

### iii-AFM

**Figure 3:**
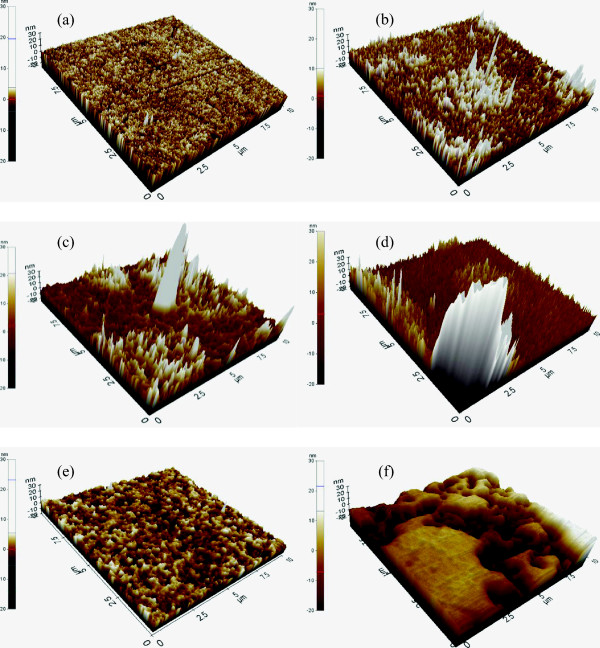
**Surface topography of ZnO and ZnO:Cs**_**2**_**CO**_**3 **_**films on ITO.** AFM images of **(a)** ZnO, **(b)** ZnO:Cs_2_CO_3_ (3:1), **(c)** ZnO:Cs_2_CO_3_ (2:1), **(d)** ZnO:Cs_2_CO_3_ (1:1), **(e)** ZnO:Cs_2_CO_3_ (1:2), and **(f)** ZnO:Cs_2_CO_3_ (1:3).

To further characterize the formation of interface modification, atomic force microscopy imaging is performed. Figure 3 illustrates the surface topography of ZnO and ZnO:Cs_2_CO_3_ films on ITO. As shown in Figure 3a, neat ZnO exhibits a smooth surface with a root mean square (RMS) roughness of 2 nm. The image of the ZnO surface was somewhat variable. This is most likely due to the fact that the sol-gel process results in a fine-grained polycrystalline film with an exposed crystal surface having various different orientations. On the other hand, some informative distinctions were observed optically, where the interface modification could be seen (Figure 3b,c,d,e,f). The interface modification by ZnO:Cs_2_CO_3_ layer (Figure 3b) shows a slightly higher RMS roughness. The RMS roughness of the modified surface (3:1) is 4.7 nm, which is more than twice that of the neat ZnO (Figure 3a). The roughness becomes higher as the blend ratio changes from 3:1 to 2:1, leading to RMS roughness of 9.5 nm (Figure 3c). However, as we can see from Figure 3d, the RMS roughness decreases to 6 nm as the blend ratio changes from 2:1 to 1:1. The lowest roughness is obtained with the blend ratio of 1:2, where the RMS roughness is around 2.75 nm (Figure 3e). As a result, the surface morphology of interface modified (1:2) demonstrates a good and smother surface. Finally, as the amount of Cs_2_CO_3_ becomes larger, the roughness gets higher. This can be seen from Figure 3f, where the RMS roughness jumps to 10.41 nm. For more information on surface topography, please see Supporting Information. From these AFM images, one finds that there is a clear hint that modified surface gives slightly rough topography. The increase in surface roughness can be correlated to better ordering of P3HT chains in blend films leading to better performing devices; nonetheless, it does not directly give quantitative information regarding crystallinity and can only be an indirect method to determine the performance of devices [52]. As we can see from Supplementary Information (Additional file 1: Figure S1), the modified interface (ZnO:Cs_2_CO_3_) with the blend of 1:1 is one of lowest RMS roughness with a pretty smooth morphology. Therefore, we have adopted 1:1 blend ratio for the entire work represented in this work.

### iv-Transmittance, Raman, XRD, and PL

**Figure 4:**
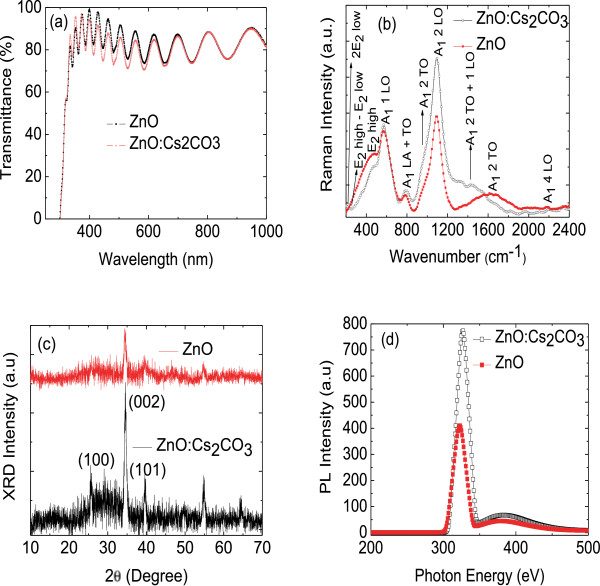
**Transmittance spectra, Raman spectra, XRD intensity, and PL intensity of ZnO and ZnO:Cs**_**2**_**CO**_**3. **_**(a)** Transmittance spectra, **(b)** Raman spectra, **(c)** XRD intensity, and **(d)** PL intensity of ZnO and ZnO:Cs_2_CO_3_ layers coated on ITO substrate.

Figure 4a depicts the room temperature transmittance spectra of ZnO and ZnO:Cs_2_CO_3_ thin films. It can be seen that the average transparency in the visible region is 83% for the ZnO layer but decreases with the presence of Cs_2_CO_3_. The average transmittance of ZnO:Cs_2_CO_3_ is 79%, and the average calculated optical bandgap for ZnO and ZnO:Cs_2_CO_3_ is 3.25 and 3.28 eV, respectively. The quantum confinement size effect (QSE) usually takes place when the crystalline size of ZnO is comparable to its Bohr exciton radius. Such size dependence of the optical bandgap can be identified in the QSE regime when crystalline size of ZnO is smaller than 5 nm [53,20]. In addition, Burstein-Moss effects can be used to deduce the increase in the optical bandgap. The Burstein-Moss effects demonstrate that a certain amount of extra energy is required to excite valence electron to higher states in the conduction band since a doubly occupied state is restricted by the Pauli principle, which causes the enlargement of the optical bandgap [54]. Therefore, the enlargement in the optical bandgap is caused by the presence of excess donor electrons, which is caused by alkali metals situated at interstitial sites in the ZnO matrix [55].

Figure 4b presents the room-temperature (RT) Raman spectra of the ZnO and ZnO:Cs_2_CO_3_ in the spectral range 200 to 1,500 cm^−1^. Raman active modes of around 322 cm^−1^ can be assigned to the multiphonon process *E*_2_ (high) to *E*_2_ (low). The second order *E*_2_ (low) at around 208 cm^−1^ is detected due to the substitution of the Cs atom on the Zn site in the lattice. The strong shoulder peak at about 443 cm^−1^ corresponds to the *E*_2_ (high) mode of ZnO, which *E*_2_ (high) is a Raman active mode in the wurtzite crystal structure. The strong shoulder peak of *E*_2_ (high) mode indicates very good crystallinity [56]. For the ZnO:Cs_2_CO_3_ layer, one additional and disappearance peaks has been detected in the Raman spectra. The additional peak could be assigned to the combination modes such as *A*_1_(2 TO + 1 LO), while this diminished peak can be assigned to *A*_1_(2 TO) as shown in Figure 4b [57]. From these observations, we conclude that doping can be considered to be the main factor that would cause the lattice distortion of the crystals, for it is usually different from the atomic radius of different elements. As the ZnO is doped with Cs_2_CO_3_, the shoulder peak position (the *E*_2_ (high) mode) shifts to 435 cm^−1^ from 433 cm^−1^ as shown in Figure 4b.

Figure 4c shows the XRD patterns of the ZnO and ZnO:Cs_2_CO_3_ thin films deposited on ITO substrates. It is found that the ZnO and ZnO:Cs_2_CO_3_ thin layers show peaks corresponding to (100), (002), and (101) planes. All detected peaks match the reported values of the hexagonal ZnO structure with lattice constants *a* = 3*.*2374 Å and *c* = 5*.*1823 Å; the ratio *c*/*a* ~1.60 and this value is indeed in agreement with the ideal value for a hexagonal cell (1.633). The intensity of the peak corresponding to the (002) plane is much stronger than that of the (100) and (101) plane in the pure ZnO as well as ZnO:Cs_2_CO_3_ layers. This suggests that the *c* axis of the grains become uniformly perpendicular to the substrate surface. The XRD pattern of ZnO:Cs_2_CO_3_ layer is dominated by the (002) plane, with very high intensity. The highest intensity of the XRD peaks obtained from ZnO:Cs_2_CO_3_ film indicates a better crystal quality. One possible reason for such a high intensity is probably the possibility of heterogeneous nucleation, which is facilitated with the presence of Cs ions in the ZnO structure. It is evident that as the Cs_2_CO_3_ doping concentration increases, the lattice parameters ‘*a*’ and ‘*c*’ slightly increase (data not shown).

Figure 4d shows the PL spectra of the ZnO and ZnO:Cs_2_CO_3_ films excited by 325-nm Xe light at room temperature. The PL spectra of ZnO contain a strong UV band peak at 326 nm and a weak and broad green band located from 400 to 450 nm. The UV emission peak is originated from excitonic recombination, which is related to the near-band-edge emission of ZnO. Additional weak broad green peak located from 400 to 450 nm refers to a deep-level or trap state emission. The green transition is designated to the singly ionized oxygen vacancy in ZnO and the emission results from the radiative recombination of electron occupying the oxygen vacancy with the photo-generated hole [58]. The strong UV and weak broad green bands imply good crystal surface. The blue shift of the UV emission peak position of ZnO:Cs_2_CO_3_ (330 nm) thin film with respect to the ZnO layer is probably caused by the band-filling effect of free carriers. A strong quenching of the UV emissions also indicates that the crystalline ZnO:Cs_2_CO_3_ layer contains a large numbers of defects that can trap photogenerated free electron and/or holes.

Table 1 tabulates the electrical resistivity of ZnO and ZnO:Cs_2_CO_3_ thin films. As shown in Table 1, the resistivity increased from 2.2 × 10^−3^ to 5.7 × 10^−2^ ohm cm. ZnO is known as an *n*-type metal-oxide semiconductor due to the excess Zn or O vacancies. When Cs_2_CO_3_ is doped with ZnO, the interstitial Cs atoms decrease the number of interstitial Zn atoms. The presence of monovalent Cs in Zn site basically creates a hole, which tends to form *p*-type conduction. The decrease in the number of interstitial Zn atoms and/or the reduction of O vacancies is the reason for the increment in resistivity of ZnO:Cs_2_CO_3_ films.

**Table 1:** **Lattice parameters, FWHM, and grain size of ZnO and ZnO:Cs**_**2**_**CO**_**3**_

**Thin film**	***a *** **(Å)**	***c *** **(Å)**	**2θ (degree)**	**FWHM (degree)**	**Grain size (nm)**	**Resistivity (ohm cm)**
ZnO	3.2374	5.1823	34.589	0.220	66	2.2 × 10^−3^
ZnO:Cs_2_CO_3_	3.2382	5.1835	34.601	0.146	99.46	5.7 × 10^−2^

### v-J-V, EQE, and stability characteristics

**Figure 5:**
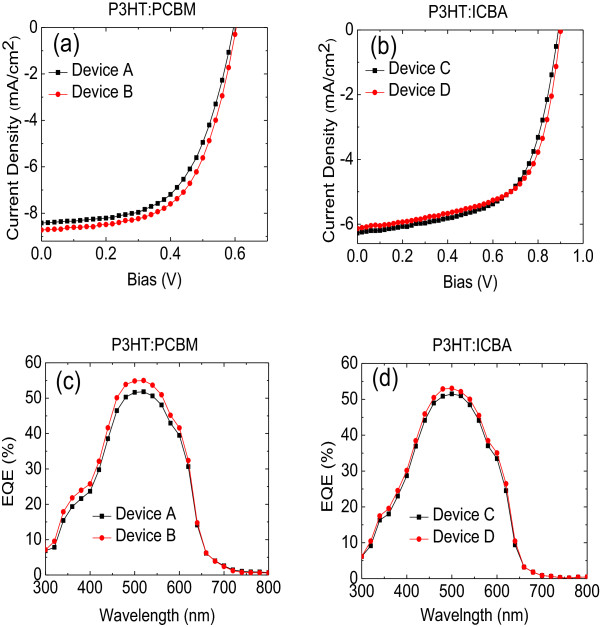
**J-V characteristics of P3HT:PCBM- and P3HT:ICBA-based devices. (a)** Device A (ZnO and PEDOT:PSS), device B (ZnO:Cs_2_CO_3_ and PEDOT:PSS), and **(b)** device C (ZnO and PEDOT:PSS), device D (ZnO:Cs_2_CO_3_ and PEDOT:PSS). External quantum efficiency of P3HT:PCBM and P3HT:ICBA-based devices; **(c)** device A (ZnO and PEDOT:PSS), device B (ZnO:Cs_2_CO_3_ and PEDOT:PSS), and **(d)** device C (ZnO and PEDOT:PSS), device D (ZnO:Cs_2_CO_3_ and PEDOT:PSS).

Figure 5a shows the J-V characteristics for P3HT:PCBM-based devices with different electron and hole buffer layers: ZnO and PEDOT:PSS (device A) and ZnO:Cs_2_CO_3_ and PEDOT:PSS (device B (Figure 5a)). As we can see from the device B with ZnO and PEDOT:PSS as electron and hole buffer layers, respectively, the short-circuit current density (J_sc_) is 8.42 mA/cm^2^; open-circuit voltage (V_oc_) is 0.60 V; and fill factor (FF) is 57.7%, along with power conversion efficiency (PCE) of about 2.89%. As we introduced Cs_2_CO_3_ to the ZnO film (device B), the J_sc_, and FF increase slightly to 8.72 mA/cm^2^ and 59.3%, respectively. However, the V_oc_ remains unchanged. The increments in J_sc_ and FF lead to an improvement in PCE to 3.12%. The improved J_sc_ can be attributed to interface modification by removing the trap states at the interface of the ZnO. When the surface of ZnO is modified with this dipole, the average conversion efficiency is further improved by 8% compared to devices without this dipole. Meanwhile, the improved FF can be attributed to the dipole on the Cs_2_CO_3_, which helps to enhance charge selectivity and reduce the charge recombination losses at the interface. It is worth to note that as the FF increases from device A to device B, the R_s_ decreases to lower values, where the R_s_ for devices A and B is 1,333 and 1,176 ohm cm^2^, respectively. This indicates that the interface modification reduces the R_s_ of the device. The series and shunt resistances are determined from the inverse gradient of the J-V curve at 1 V and at the short-circuit current density under illumination.

An important issue is to check whether the work function shifts are also reflected in the performance of devices when other active materials are used. As demonstrated by several authors, if one employs interface modification into any device, it should, in principle, be possible to see some positive improvements as a result of the interface modification, regardless of active materials used. For this purpose, we made another set of inverted solar cells based on P3HT:ICBA with the configuration ITO/ZnO:Cs_2_CO_3_/P3HT:ICBA/PEDOT:PSS/Al. As a reference, solar cells without interface modification were also fabricated.

Figure 5b shows the J-V characteristics of P3HT:ICBA-based devices with two different structures. As mentioned in the ‘Experimental’ Section, these two different types of structures are ITO/ZnO/P3HT:ICBA/PEDOT:PSS/Al-device C and ITO/ZnO:Cs_2_CO_3_/P3HT:ICBA/PEDOT:PSS/Al-device D. As expected, the effective dipole moment created by interface modification shifted the work function of the ITO electrode by nearly 1 eV, thereby reducing the electron injection and improving the ohmic contact for electron injection with P3HT:ICBA. The inverted solar cells (device A) exhibited a contact energy barrier of typically 2.1 eV due to the work function of ITO (4.8 eV), resulting in J_sc_ that was slightly lower. As observed in Figure 5b, the reference device exhibits J_sc_, V_oc_, FF, and PCE of about 6.28 mA/cm^2^, 0.89 V, 60.7%, and 3.40%, respectively. The calculated R_s_ for this device is around 6,666 ohm. For device D, the PCE increases from 3.40 to 3.43%. This 0.88% increment in the PCE is attributed to the improvement in FF, where the FF increases from 57.7 to 59.3%. A similar trend in R_s_ can also be seen in P3HT:ICBA-based device, where the R_s_ decreases together with the increment of FF.In addition, the performance of inverted solar cells in terms of external quantum efficiency (P3HT:PCBM-based devices) is shown in Figure 5c. Basically, the EQE is defined as the ratio between the generated charge carriers and the incident photons. Device A shows a maximum EQE value of ~51.80% at the absorption wavelength of ~520 nm. However, the EQE of device B has outperformed the EQEs of device A, in which it exhibits a maximum of about 55% at ~520 nm of absorption wavelength.

The external quantum efficiency of the P3HT:ICBA-based devices with the inverted device geometries are shown in Figure 5d. For inverted reference solar cells (device C), the maximum EQE is 51.51% at 500 nm, where the EQE of device D is 53.05%. These results (device B and device D) further shed the light that the improvement in devices performances is related to interface modification which has modified the work function of the ITO electrode. As mentioned above, the presence of Cs_2_CO_3_ have improved the surface area of ZnO:Cs_2_CO_3_ and PEDOT:PSS through the good interfacial contact between ZnO:Cs_2_CO_3_ layer and ITO layer, and PEDOT:PSS and Al layer, leading to the considerably high EQE.

In order to get a better understanding on the stability and lifetime of all fabricated inverted solar cells, we kept all devices in air under ambient condition according to the previously reported ISOS-L-1 procedure [43]. All stability measurements were performed in the presence of air and the stability of these devices were periodically measured weekly up to 4 weeks. Table 2 shows the evolutions (device A) of J_sc_, V_oc_, FF, and PCE over 4 weeks (see Additional file 2: Figure S2a). All obtained values were averaged over four different cells in the same sample. After 1 week of storage, PCE of device A deteriorated by 5.19% from its original value. This deterioration is due to the losses in FF of about 6.24% to 54.1%. The decrement in FF is accompanied with the increment in R_s_, in which the R_s_ of the fresh device is 1,333 ohm cm^2^, while the R_s_ after 1 week 1,539 ohm. However, the V_oc_ remained stable, while the J_sc_ increases slightly to 8.60 mA/cm^2^. However, as we blended Cs_2_CO_3_ together with ZnO (Table 3), we observed a significant improvement in the stability of the device. After 4 weeks of ambient storage, both the J_sc_ and FF dropped by 3.33 and 7.08%, respectively, leading to 11.2% reduction in PCE (see Additional file 2: Figure S2b). From the stability measurements, devices B and D outperformed devices A and C, where device A was completely dead by the second weeks.

**Table 2:** Environmental degradation parameters of P3HT:PCBM-based devices (ZnO and PEDOT:PSS-device A)

**Device A**	**J**_**sc **_**(mA/cm**^**2**^**)**	**V**_**oc **_**(V)**	**FF (%)**	**PCE**
Original	8.42	0.60	57.7	2.89
Week 1	8.60	0.59	54.1	2.74

**Table 3:** **Environmental degradation parameters of P3HT:PCBM-based devices (ZnO:Cs**_**2**_**CO**_**3 **_**and PEDOT:PSS-device B)**

**Device B**	**J**_**sc **_**(mA/cm**^**2**^**)**	**V**_**oc **_**(V)**	**FF (%)**	**PCE**
Original	8.72	0.60	59.3	3.12
Week 1	8.17	0.60	58.7	2.86
Week 2	8.20	0.60	57.9	2.83
Week 3	8.47	0.60	57.0	2.88
Week 4	8.43	0.60	55.1	2.77

It is interesting to see how P3HT:ICBA-based devices behave during 4 weeks of stability and lifetime measurements. The stability study for P3HT:ICBA-based devices are similar to the abovementioned measurements, and all parameters were averaged over four different cells in the same sample. As we can see from Table 4 (device C), after 4 weeks of stability tests, the performance of these devices is deteriorated by 10.3% of its initial value (see Additional file 2: Figure S2c). This is due to the fact that there are losses in all parameters: J_sc_, V_oc_, and FF. As for device D (Table 5), the performance of the inverted solar cells is slightly worse compared to that of device C, where, after 4 weeks of stability measurements, the PCE of device C decreases to 3.01%, which is about 12.3% drop from its original value (see Additional file 2: Figure S2d). The deterioration of device D is comparable to the deterioration of device C although all parameters in device D experienced a slightly bigger reduction from their initial values. The J_sc_, V_oc_, and FF suffer 8.63, 0.24, and 1.77% reduction from their original values, respectively.

**Table 4:** Environmental degradation parameters of P3HT:ICBA-based devices (ZnO and PEDOT:PSS-device C)

**Device C**	**J**_**sc **_**(mA/cm**^**2**^**)**	**V**_**oc **_**(V)**	**FF (%)**	**PCE**
Original	6.28	0.89	60.7	3.40
Week 1	6.01	0.89	59.5	3.16
Week 2	5.92	0.88	59.8	3.13
Week 3	5.75	0.88	58.8	2.97
Week 4	6.12	0.88	57.0	3.05

**Table 5:** **Environmental degradation parameters of P3HT:ICBA-based devices (ZnO:Cs**_**2**_**CO**_**3 **_**and PEDOT:PSS-device D)**

**Device D**	**J**_**sc **_**(mA/cm**^**2**^**)**	**V**_**oc **_**(V)**	**FF (%)**	**PCE**
Original	6.14	0.90	62.0	3.43
Week 1	5.89	0.90	61.1	3.22
Week 2	5.69	0.89	60.9	3.08
Week 3	5.42	0.87	59.0	2.79
Week 4	5.61	0.88	60.9	3.01

## Conclusion

In conclusion, we have found that modification of the interface between the inorganic ITO and photoactive layer can improve the performance of inverted solar cells. The modification of ITO leads to 8% improvement over unmodified ITO inverted devices. This interface modification serves multiple functions that affect the photoinduced charge transfer at the interface, which include the reduction the recombination of charges, passivation of inorganic surface trap states, and improvement of the exciton dissociation efficiency at the polymer/ZnO interface. Moreover, the stability of these modified devices is slightly better compared with unmodified ones.

## Competing interests

The authors declare that they have no competing interests.

## Authors' contributions

HPK carried out all electrical measurements; ARBMY designed the study and drafted the manuscript; SJL, HJL, HMK, GJS, and JHY performed XPS and UPS, AFM, XRD, and Raman, photoluminescence, and transmittance, respectively; and ARBMY and JJ finalized the final manuscript. All authors read and approved the final manuscript.

## Supplementary Material

Additional file 1: Figure S1 AFM images of ZnO and ZnO:Cs_2_CO_3_ layers with different blend ratios. Click here for file

Additional file 2: Figure S2 J-V characteristics evolutions of P3HT:PCBM- and P3HT:ICBA-based devices (a) ZnO and PEDOT:PSS-Device A, (b) ZnO:Cs_2_CO_3_ and PEDOT:PSS-Device B, (c) ZnO and PEDOT:PSS-Device C, and (d) ZnO:Cs_2_CO_3_ and PEDOT:PSS-Device D. Click here for file
