# Supplementary figures and images for: Effect of ZnO:Cs2CO3 on the performance of organic photovoltaics
Source: Nanoscale Res Lett. 2014 Jun 27;9(1):323. doi: 10.1186/1556-276X-9-323 (PMC4099096; doi:10.1186/1556-276X-9-323)

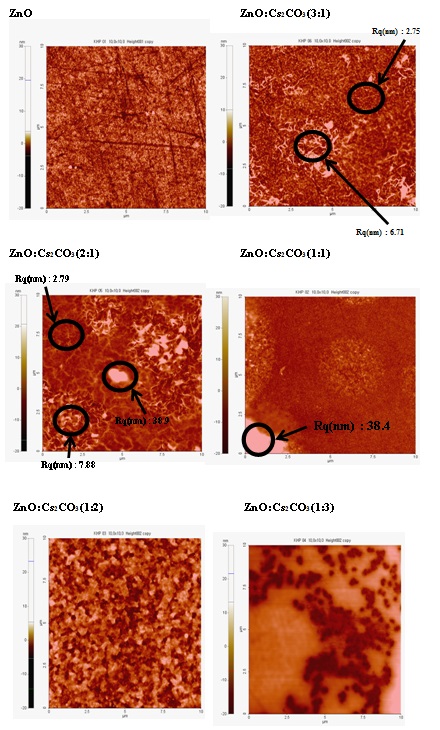

Supplement: Additional file 1: Figure S1 — AFM images of ZnO and ZnO:Cs2CO3 layers with different blend ratios. [file 1556-276X-9-323-S1.jpeg]

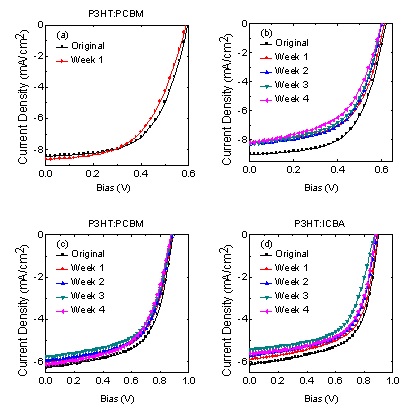

Supplement: Additional file 2: Figure S2 — J-V characteristics evolutions of P3HT:PCBM- and P3HT:ICBA-based devices (a) ZnO and PEDOT:PSS-Device A, (b) ZnO:Cs2CO3 and PEDOT:PSS-Device B, (c) ZnO and PEDOT:PSS-Device C, and (d) ZnO:Cs2CO3 and PEDOT:PSS-Device D. [file 1556-276X-9-323-S2.jpeg]
